# Vitrified embryo transfer in Merino sheep under extensive conditions

**DOI:** 10.21451/1984-3143-AR2018-0108

**Published:** 2019-10-24

**Authors:** Alejandro Gibbons, Macarena Bruno-Galarraga, Jimena Fernandez, Antonio Gonzalez-Bulnes, Marcela Cueto

**Affiliations:** 1 Laboratorio de Reproducción de Rumiantes Menores, INTA Bariloche, San Carlos de Bariloche, Argentine.; 2 Departamento de Reproducción Animal, SGIT-INIA, Madrid, Spain.

**Keywords:** Vitrification, cryosurvival, in vivo-produced embryos

## Abstract

The aim was to evaluate pregnancy success after transfer of embryos vitrified in micropipette tips in Merino sheep under extensive conditions. A second objective was to evaluate the influence of embryo stage in such pregnancy rate. One hundred and twenty-seven embryos were rewarmed and transferred into recipient ewes. On rewarming, the embryos were placed into three-step cryoprotectant dilutions. Finally, prior to transfer to recipient females, embryos were maintained in Basic Medium for 5 min at 25ºC and were re-evaluated by morphological criteria; all degenerated embryos were eliminated. Recipient ewes (n = 150) were treated for estrus with sponges placed for 14 days and 300 IU of eCG. At embryo transfer, three experimental groups were defined: morulae transferred on Day 7, blastocysts transferred on Day 7 and blastocysts transferred on Day 8 after sponge removal. In all groups, semi-laparoscopic transfer of one rewarmed embryo per recipient was performed. Pregnancy was diagnosed by ultrasonography on day 28 after embryo transfer. The embryo selection rate after rewarming was higher for blastocysts (89.3% - 67/75) compared to morulae (65.9% - 60/91) (P < 0.05). Pregnancy diagnosis showed a 38.3% (23/60) of success after morula transfer on Day 7 post progestagen removal. The day of transfer showed a significant influence on pregnancy rate after blastocyst transfer (Day 8, 55.9% - 19/34 vs Day 7, 21.2% - 7/33) (P < 0.05). Blastocysts transfer on Day 8 showed the highest global efficiency (pregnancies/total embryos after rewarming) (47.5% - 19/40) (P < 0.05). In conclusion, reproductive efficiency obtained by vitrified embryo transfer allows its recommendation for embryo transfer programs under extensive conditions. The importance of considering the synchrony between the embryo age and the recipient uterus stage is emphasized.

## Introduction

The first lamb born following transfer of a vitrified embryo was reported by Széll *et al*. (1990). Afterwards, in sheep and other species, vitrification of embryos was in the focus of intense research (Schiewe *et al*., 1991; Ali and Shelton, 1993; Brown and Radcievic, 1999) and several methodologies were developed. Among them, we can highlight the use of electron microscopy grids (Martino *et al*., 1996), fine capillaries (Vajta *et al*., 1997), cryo-loops (Lane *et al*., 1999), cryo-tops (Kuwayama and Kato, 2000), straws (Baril *et al*., 2001) and micropipette tips (Cremades *et al*., 2004; Gibbons *et al*., 2011).

However, in spite of these efforts, there is scarce transfer of the vitrification technology in sheep embryos from the bench to the field. The technique has not been extensively used because there is no standard procedure and because limited reports on fertility under field conditions are available (Green *et al*., 2009; Gibbons *et al*., 2011).

Hence, the main objective of this study was to evaluate, in Merino sheep under extensive conditions, the pregnancy success obtained after transfer of embryos which were vitrified using a technique previously developed at our laboratory (micropipettes tips; Gibbons *et al*., 2011). A second objective was to evaluate the influence of embryo stage at vitrification (morula or blastocyst) in such pregnancy rate, since previous studies in classical freezing protocols have addressed a lower survival of morulae to cryopreservation (Garcia-Garcia *et al*., 2006).

## Materials and methods

Ethical concerns were taken into account by adhering to local animal welfare regulations and practices. The present study complies with national and international standards regarding the use of animals for experimentation and it has been approved under the rules of the Regional Institutional Committee for the Care and Use of Animals of Experimentation (CICUAE-PATNOR INTA, Disposition 066/17 – Resolution 533/16).

A total of 174 multiparous Merino sheep, 4-6 years old were used in the experiment, 24 ewes were used as embryo donors and 150 ewes were used as embryo recipients. The experiment was conducted during the natural breeding season (May-Jun, HS) in two locations. Embryo donors were kept at the Experimental Station of the Instituto Nacional de Tecnología Agropecuaria (INTA), Bariloche, Río Negro state, Argentine (latitude 41º 07´ 23” S, longitude 71º 15´ 12” W), under natural day-length. Animals were kept under intensive management and received 1.300 g/ewe/day of concentrate (homemade mixture with 18% of crude protein) for having a moderate body condition (2.7 ± 0.1, subjective scale, 1 emaciated, 5 obese) (Russel *et al*., 1969). Water was provided ad libitum.

Embryo recipients were kept at the Experimental Farm of the INTA, Bariloche, Río Negro (41º 7’ 23” S, 70° 43’ 12” W), under natural grazing on pastures and with free access to water. Body condition score was 2.6 ± 0.0. The paddock had an area of 243.8 hectares dominated by a grassy shrub steppe with a forage production of 100 kg/ha/year.

Briefly, the study design consisted on the recovery of embryos from donor sheep on days 7 and 8 after sponge removal (Section Superovulation, artificial insemination and embryo recovery in donor ewes). Immediately after recovery, embryos were assessed, vitrified and then rewarmed (Section Assessment, vitrification and rewarming of embryos). At embryo transfer, three experimental groups of recipient ewes were defined: morulae transferred on Day 7, blastocysts transferred on Day 7 and blastocysts transferred on Day 8 (Section Synchronization of estrous cycle, embryo transfer and pregnancy diagnosis in recipient ewes). Detailed procedures are presented in the next sections.

### Superovulation, artificial insemination and embryo recovery in donor ewes

The estrous cycle of embryo donors was synchronized by the insertion of intravaginal progestagen sponges (60 mg of medroxyprogesterone acetate, MAP; Progespon^®^, Syntex, Argentine), for 14 days (Day 0: day of sponge insertion). The superovulatory treatment, adapted from Gibbons *et al*. (2010), consisted of the administration of a reduced dose of 80-mg pFSH in six decreasing doses (18 mg x 2; 14 mg x 2; 8 mg x 2, i.m.; Folltropin-V^®^, Bioniche, Canada), injected twice daily from the morning of Day 12 to 12 h after pessary removal. A single dose of equine Chorionic Gonadotropin (200 IU of eCG, i.m.; Novormon^®^, Syntex, Argentine) was administered concomitantly with the fifth FSH dose and the pessary removal.

Estrus detection was performed with vasectomized adult rams, twice daily (8 a.m. and 8 p.m.) from 24 to 60 h after pessary removal. Twelve hours after estrus detection, ewes were artificially inseminated with 100 million frozen-thawed spermatozoa from the same batch and ram (post-thaw progressive forward motility 40-45%). Half of each semen dose was introduced into each uterine horn by laparoscopic method, using a cannula for intrauterine artificial insemination (Aspic for ewe insemination, 23-G needle, IMV^®^, L’Aigle, France).

Embryo recovery was performed by surgical laparotomy on Days 7 (n = 12) and 8 (n = 12) after progestagen removal for obtaining morulae and blastocysts. Females fasted 24 h prior, were anaesthetized with xylazine (0.2 mg/kg, i.m.; Kensol^®^ 2%, Konig, Argentine) and ketamine hydrochloride (2.5 mg/kg, i.m.; Ketalar^®^, Parke-Davis, Argentine). In addition, a local anesthetic (lidocaine, 1ml, i.m.; Frankaina^®^ 2%; Fatro Von Franken, Argentine) was administered at the site of the surgery on the midline of the abdomen. In brief, laparotomy was performed flushing each uterine horn with 20 ml of commercial embryo recovery medium (Vigro Complete Flush^®^, Bioniche, USA), pre-warmed to 38ºC and supplemented with 10% adult bovine serum (Internegocios^®^, Argentine). Embryo recovery medium was injected by a sterile syringe with an 18-G blunt needle, inserted close to the uterine horn bifurcation and directed from the uterine horn toward the utero-tubal junction, where a catheter was attached with silk (3/0, Ethicon^®^, Brazil). The catheter consisted of a pediatric nasogastric tube (k33) fastened to a blunt needle (50/20), with a central opening at the tip and two lateral openings. Once embryo recovery was finished, surgical incisions were closed by suture. General antibiotic was administered in the form of oxytetracycline (1 ml/10 kg im), and local antibiotic (gentamicine) was applied at the site of the abdominal incision. Immediately after embryo recovery, a single IM injection of 125-μg Cloprostenol (Estrumate®, Intervet Schering Plough, Argentine) was administered to induce corpora lutea (CL) regression.

### Assessment, vitrification and rewarming of embryos

All embryos were kept at 25°C in holding solution (Syngro^®^, Bioniche, USA) and evaluated just after recovery by morphological criteria (IETS, 1998), using a stereomicroscope (Olympus SZ, Olympus Optical Co., LTA., Japan); only embryos in morula and blastocyst stages classified as grade 1 (excellent) or grade 2 (good) were selected for vitrification in micropipette tips, using the methodology described by Gibbons *et al*. (2011).

Vitrification and rewarming procedures were performed using a basic medium (BM) comprising commercial flushing medium (Bovi-pro^®^, Minitube, USA) supplemented with 20% fetal bovine serum (FBS^®^, Internegocios, Argentine). Other chemicals used for embryo vitrification and rewarming were purchased from Sigma Chemical Co. (St. Louis, MO, USA).

For vitrification, briefly, all embryos were exposed to three different solutions at room temperature, according to the following procedure: (i) BM + 10% glycerol (G) for 5 min, (ii) BM + 10% G + 20% ethylene glycol (EG) for 5 min and (iii) BM + 25% G + 25% EG (vitrification solution) for 30 s. Embryos were then aspirated using an automatic 10±0.1 µl micropipette (Eppendorf, USA) and loaded in 1.5 µl vitrification solution into the lumen of plastic tips (2 embryos/tip; 10 ± 0.1 µl micropipette tips, Eppendorf Inc., USA). After removal from the micropipette, tips were introduced into 3.6 ml cryo-tubes (Nunc, Denmark) which were filled with liquid nitrogen and stored in a liquid nitrogen container for one month prior rewarming.

On rewarming, the micropipette tips were warmed between the thumb and middle fingers for 10 s and then embryos were immersed in different solutions at 25ºC in three dilution steps for 5 min each, to allow the removal of the intracellular cryoprotectant: i) BM + 12.5% G + 12.5% EG + 0.5M sucrose; ii) BM + 0.5M sucrose and iii) BM + 0.25M sucrose. Finally, prior to transfer to recipient females, embryos were maintained in BM for 5 min at 25ºC and were re-evaluated by morphological criteria; all degenerated embryos were eliminated (D´Alessandro and Martemucci, 2016).

### Synchronization of estrous cycle, embryo transfer and pregnancy diagnosis in recipient ewes

The estrous cycle of the 150 recipient Merino ewes were synchronized by the insertion of intravaginal progestagen sponges (60 mg of medroxyprogesterone acetate, MAP; Progespon^®^, Syntex, Argentine) for 14 days plus a dose of eCG (300 IU of eCG, i.m.; Novormon^®^, Syntex, Argentine) at progestagen removal.

At embryo transfer, three experimental groups were defined by combining embryo stage and day after progestagen removal in the recipients: morulae transferred on Day 7 (Morula-TD_7_ Group; n = 60 embryos), blastocysts transferred on Day 7 (Blastocyst-TD_7_ Group; n = 33 embryos) and blastocysts transferred on Day 8 (Blastocyst-TD_8_ Group; n = 34 embryos). Because it was decided to assign the largest number of embryos to each of the experimental groups, the Morula-TD_8_ group was not constituted. Transfer procedure was carried out under general and local anesthesia and using antibiotic administration described in last section in all groups.

Firstly, the presence of at least one CL was assessed by laparoscopy. In the responding ewes, a small laparotomy (around 1 cm) was made on the midline of the abdomen, cranial to the udder. The end of the uterine horn corresponding to the ovary bearing the CL was exposed using a non-traumatic clamp and, following its puncture with a 18-G needle, one embryo per recipient (Green *et al*., 2009) was placed into the lumen of the uterine horn by using a piston pipette for embryo transfer (Assipettor^®^, Minitüb, Germany). The uterine horn was then allowed to return into the abdomen and laparotomy was closed by suture. All embryo transfers were performed within 30 min after cryoprotectant removal.

On Day 28 after embryo transfer, pregnancy diagnosis was performed by transrectal ultrasonography with a 5MHz linear array transducer (Aloka 500SSD, Japan), and pregnant ewes were allowed to lamb naturally.

### Statistical analysis

This study was designed to compare the reproductive success of embryo transfer in sheep, taking into account the stage of embryo development and day of embryo transfer after progestagen removal. Data analysis was carried out using the CATMOD procedure of SAS (SAS, 2003). CATMOD analyzes categorical data using a linear model similar to ANOVA. Statistical significance was accepted from P < 0.05. Results were expressed as the mean ± standard error.

## Results

A total of 189 embryos were recovered from the 24 donor ewes (7.9±1.0 embryos/ewe) on day 7 (46.6% - 88/189) and day 8 (53.4% - 101/189) after pessary removal. Of these 189 embryos, 166 were selected as grades 1 and 2 for vitrification (87.8%), 91 morulae (54.8% - 91/166) and 75 blastocysts (45.2% - 75/166).

After rewarming, a total of 127 embryos were found to be morphologically normal for transfer (76.5%; 127/166, transferred embryos/vitrified embryos), carrying out a total of 127 embryo transfers in recipient females.

When the presence of at least one CL was evaluated by laparoscopy, a total of 13 ewes were discarded because of the absence of CL. Ten recipient ewes were not used because there were no available embryos.

The embryo selection rate post rewarming was higher for blastocysts compared to morulae (89.3% – 67/75 vs 65.9% - 60/91; P < 0.05).

Pregnancy diagnosis showed a 38.3% of success after morula transfer on Day 7 after progestagen removal (Table 1). While day of transfer showed a significant influence on yields obtained after blastocyst transfer (55.9% of pregnancies in the Blastocyst-TD_8_ Group vs 21.2% in the Blastocyst-TD_7_ Group; P < 0.05).

**Table 1:** Reproductive efficiency of ovine embryos vitrified and transferred according to embryo stage and day after progestagen removal in recipient ewes.

Group1	Selection rate after rewarming2 (%)	Recipients3 (n)	Pregnancy rate (%)	Global efficiency4 (%)
Morula-TD_7_	60/91 (65.9)^a^	60	23/60 (38.3)^ab^	23/91 (25.3)^a^
Blastocyst-TD_7_	33/35 (94.3)^b^	33	7/33 (21.2)^a^	7/35 (20.0)^a^
Blastocyst-TD_8_	34/40 (85.0)^b^	34	19/34 (55.9)^b^	19/40 (47.5)^b^

a, b : Different letters in the same column indicate P < 0.05 (CATMOD procedure, SAS 2003); ^1^Morula-TD_7_, morulae transferred on Day 7; Blastocyst-TD_7_, blastocysts transferred on Day 7; Blastocyst-TD_8_, blastocysts transferred on Day 8; ^2^Selected embryos/Total embryos after rewarming; ^3^Only transferred recipients; ^4^Pregnancies/Total embryos after rewarming.

When considering the total number of pregnancies in relation to the total number of rewarmed embryos, Blastocyst-TD_8_ Group (47.5%) showed the highest global efficiency in relation to the Morula-TD_7_ (25.3%) and Blastocyst-TD_7_ Groups (20.0%) (P < 0.05).

All pregnancies were carried to term and no post partum mortality was observed.

## Discussion

The results of the present study indicate that vitrification/rewarming procedure is a reliable method to be applied in sheep maintained under extensive rearing, but conditioning factors derived from embryo stage and day of transfer should be considered.

Embryo stage was firstly critical for survival rate after vitrification/rewarming and, overall, blastocysts showed better percentages of cryotolerance than morulae (89.3 vs 65.9%). These percentages are similar to previously reported for morulae (70%; Songsasen *et al*., 1995) and blastocysts (84%; Dattena *et al*., 2000). However, Gibbons *et al*. (2011) obtained similar selection rates of transferable embryos after rewarming, independently of embryo stage (89.5 and 85.7% for morulae and blastocysts, respectively). The relatively low survival rate in morulae after vitrification/rewarming in relation to blastocysts indicates that the blastocyst stage has a higher survival to vitrification procedures, in agreement with Garcia-Garcia *et al*. (2006), who indicated that survival rates following conventional freezing improve as embryo stage progresses.

Embryo stage was also critical for pregnancy success, although modulated by the day of estrous cycle of the recipient at embryo transfer. The pregnancy rate obtained after transfer of embryos at morula stage on Day 7 after progestagen removal was around 40%, providing evidence for the hypothesis that vitrification could be an alternative to increase viability in early stages of embryo development (Vajta, 2000). In addition, cryotolerance rate for the vitrification/rewarming procedure was higher (65.9%) than for classical freezing (46.3% reported by Garcia-Garcia *et al*., 2006).

The pregnancy rate after blastocyst transfer was strongly affected by day of transfer, being higher for blastocysts transferred on Day 8 than for blastocysts transferred on Day 7 after progestagen removal, in agreement with previous data on vitrified embryos in sheep (Naitana *et al*., 1995). These data support hypotheses addressing the critical role of an accurate synchrony between the embryo stage and the estrous cycle of the recipient female, and questioning the adequacy of blastocyst transfer at a fixed time of 7 days after sponge removal and eCG administration.

The need of synchrony between the embryo stage and the day of the estrous cycle was early postulated by Rowson and Moor (1966) for embryo transfer in the bovine species and, afterwards, data in cattle and sheep indicated that a close synchronization between embryo age and uterine stage of the recipient is a prerequisite for normal development of transferred embryos. In cattle, it has been reported that the transfer of a day-7 fresh embryo to a day-5 uterus or a day-8 uterus conditions pregnancy rate (Randi *et al*., 2016). In sheep, results in recipient ewes after the transfer of vitrified blastocysts on Days 6, 7 and 8 after estrus detection (days 7, 8 and 9 after sponge removal) were 72.7, 90.0 and 54.5% for pregnancy rates and 72.7, 80.0 and 45.5% for lambing rates (Naitana *et al*., 1995).

The results of the present study, when transferring morulae on Day 7 or blastocysts on Day 8, are similar to previously obtained in terms of both survival (50%; Baril *et al*., 2001) and pregnancy rates (55.8%; Green *et al*., 2009), but lower than those reported by other authors (62.9% lambing rate; Dattena *et al*., 2000); although we have to note that these last authors only transferred blastocysts that re-expanded after vitrification/rewarming.

Our results confirm that blastocyst transfer on Day 8 after progestagen removal is related to higher cryosurvival and pregnancy rates, as previously reported (Széll *et al*., 1990; Mc Ginnis and Youngs, 1990; Garcia-Garcia *et al*., 2005). Overall, global efficiency after blastocyst transfer on Day 8 after pessary removal (47.5%), indicates that embryo viability following cryopreservation improves as embryo developmental stage progresses. On the other hand, although rates of selection and pregnancy after vitrified morula transfer are acceptable, when considering both rates together, it is necessary to point out that a low global efficiency is observed (25.3%).

Concomitantly, using fresh embryos, Rizzo *et al*. (2012) reported that best yields were obtained when transferring two blastocysts to recipient ewes with more than two corpora lutea. However, in our study, only one embryo was transferred by recipient, and pregnancy rates were similar to that obtained by transferring two embryos per recipient female in the same experimental conditions (around 41 and 50% viable fetuses/transferred embryos for morulae and blastocysts, respectively; Gibbons *et al*., 2011). This finding is of great interest due to the higher perinatal mortality of twins when compared to single lambs under extensive production conditions (75 and 90% of lamb survival for twin and single lambs, respectively, Fernandez Abella, 2015), which would penalize the final yields obtained by the transfer of two embryos.

In conclusion, the acceptable reproductive efficiency obtained by transferring morulae on Day 7 or blastocysts on Day 8 after progestagen removal, at a proportion of one embryo per recipient ewe, allows recommending the use of this technique for embryo transfer programs performed under extensive field conditions.

The highest pregnancy rate in relation to the total number of rewarmed embryos was achieved by transferring embryos in the blastocyst stage on Day 8 after progestagen removal, such findings emphasize the importance of considering the synchrony between the age of the embryo and the stage of the recipient uterus.
